# Development of a Multiplex Droplet Digital PCR Assay for Detection of Tick-Borne Pathogens

**DOI:** 10.3390/pathogens15020212

**Published:** 2026-02-13

**Authors:** Lijuan He, Lin Zhang, Like Duan, Xuexia Hou, Jingwen Li, Qin Hao

**Affiliations:** National Key Laboratory of Intelligent Tracking and Forecasting for Infectious Diseases, Department of Spirochetosis Control, National Institute for Communicable Disease Control and Prevention, Chinese Center for Disease Control and Prevention, Beijing 102206, China

**Keywords:** *Borrelia burgdorferi sensu lato*, *Borrelia miyamotoi*, *Coxiella burnetii*, multiplex droplet digital PCR, spotted fever group Rickettsia

## Abstract

Tick-borne pathogens pose a significant threat to human health. In this study, a multiple droplet digital PCR (ddPCR) assay was developed to detect four tick-borne pathogens: *Borrelia burgdorferi sensu lato* (*Bbsl*), *Coxiella burnetii* (*C. burnetii*), spotted fever group Rickettsia (SFGR), and *Borrelia miyamotoi* (*B. miyamotoi*). Based on the singleplex ddPCR reaction system of *Bbsl*, the primer probes of the other three species were incorporated to develop a multiplex ddPCR reaction system. The annealing temperature and the final concentration of the primer probes were then optimized for multiplex ddPCR. The multiplex ddPCR assay was assessed for its sensitivity, specificity, repeatability, and ability to detect simulated and actual samples. The developed multiplex ddPCR approach enables the simultaneous detection of *Bbsl*, *C. burnetii*, SFGR, and *B. miyamotoi*. The positive target microtitre clusters are closely grouped and distinctly separated from each other, with the multiplex ddPCR assay demonstrating a dynamic range of five orders of magnitude. The limits of detection (LOD) for the multiplex ddPCR assay were 4 copies/20 µL for *Bbsl*, 3 copies/20 µL for *C. burnetii*, 3 copies/20 µL for SFGR, and 2 copies/20 µL for *B. miyamotoi*. The assay demonstrated high specificity, with no observed cross-reactivity against non-target pathogens. Performance was validated using both spiked samples and field-collected clinical specimens. In the evaluation of 30 ticks and 30 serum samples, the ddPCR method (in both singleplex and multiplex formats) achieved higher positive detection rates for all four target pathogens compared to quantitative real-time PCR (qPCR). In addition, the detection proportions of multiplex and singleplex ddPCR were consistent. Multiplex ddPCR can detect low DNA concentrations in samples and enables the absolute quantification of *Bbsl*, *C. burnetii*, SFGR, and *B. miyamotoi*, providing a novel detection approach for the clinical diagnosis of tick-borne diseases.

## 1. Introduction

Ticks are blood-sucking arthropods that cause skin damage to their hosts through bites, and they serve as vectors for many pathogens. A total of 124 tick species have been identified in China, comprising 113 ixodid species (hard ticks) across seven genera and 11 argasid species (soft ticks) in two genera. These ticks are known to carry 29 tick-borne pathogens, including *Borrelia burgdorferi sensu lato*, *Borrelia miyamotoi*, *Coxiella burnetii*, spotted fever group Rickettsia, *Anaplasma phagocytophilum*, and *Ehrlichia chaffeensis* [1]. These pathogens are responsible for diseases such as Lyme disease, tick-borne relapsing fever, spotted fever, Q fever, human granulocytic anaplasmosis, and human monocytic ehrlichiosis. Ticks in the larval stages infest the same host communities and carry diverse pathogens. Therefore, they can be infected with multiple pathogens simultaneously, increasing the likelihood of co-transmission to humans or animals [2,3,4,5].

Laboratory methods for detecting tick-borne pathogens primarily include serological and nucleic acid detection techniques. Among them, nested PCR and quantitative real-time PCR (qPCR) are commonly used for nucleic acid detection. Compared with traditional PCR, qPCR offers relative quantification, higher detection sensitivity, and eliminates the need for post-PCR processing. However, qPCR has limitations in terms of sensitivity and accuracy when the target sequence is short and the reaction system contains considerable background sequences or inhibitors. Furthermore, qPCR is only “relatively quantitative”, which makes it unsuitable for detecting low bacterial loads in samples [6]. Thus, it is crucial to develop nucleic acid detection approaches that offer higher sensitivity and efficiency for detecting tick-borne pathogens.

Digital PCR, which was introduced in the late 1990s, enables the absolute quantification of nucleic acid molecules. It directly counts DNA molecules and performs absolute quantification of the starting samples [7]. Compared with qPCR, digital PCR offers several advantages, including independence from standard curves and amplification kinetics, the ability to detect precious samples and nucleic acids, accurate detection of mixed samples at low concentrations, and reduced interference between reaction systems. Multiplex droplet digital PCR (ddPCR) is a digital PCR method in which two or more primer pairs are incorporated into the same digital PCR reaction system, enabling the simultaneous amplification of multiple nucleic acid fragments. Compared with singleplex ddPCR, multiplex ddPCR enables accurate and reliable multitarget detection in the same PCR reaction tube. This method is particularly suitable for detecting groups of nucleic acid fragments because it reduces the need for additional resources, labor, and time by allowing multiple targets to be detected in the same reaction tube [8,9]. Multiplex ddPCR is widely used for pathogenic microbial detection [10,11,12].

However, multiplex ddPCR assays for the simultaneous detection of major tick-borne pathogens remain limited. This study aimed to develop and validate a novel multiplex ddPCR assay capable of detecting and quantifying *Bbsl*, *C. burnetii*, SFGR, and *B. miyamotoi* in a singleplex reaction, thereby improving detection efficiency and providing absolute quantification.

## 2. Materials and Methods

### 2.1. Main Materials

The bacterial genomic DNA extraction kit was from Qiagen (Hilden, Germany). The ddPCR Supermix for Probes (no dUTP), Droplet Generation Oil, Droplet Analysis Oil, DG8™ Cartridges, and the QX200 Droplet Digital™ PCR System were obtained from Bio-Rad (Hercules, CA, USA). The LightCycler^®^ 480II Real-Time PCR System was from Roche (South San Francisco, CA, USA), and the NanoDrop spectrophotometer was from Thermo Fisher Scientific (Waltham, MA, USA).

### 2.2. Primer and Probe

In this study, the *recA*, *glpQ*, *ompA*, and *com1* genes of *Bbsl*, *C. burnetii*, SFGR, and *B. miyamotoi* were selected as target genes. Table 1 presents the primer and probe information.

### 2.3. Ethics Statement

This study and its protocol were reviewed and approved by the Ethics Review Committee of the National Institute for Communicable Disease Control and Prevention (ICDC) and the Chinese Center for Disease Control and Prevention. Written informed consent was obtained from all patients.

### 2.4. Sample Collection

Tick and human clinical samples were collected concurrently for this study.

More than 300 adult *Rhipicephalus microplus* collected from cattle in Yongxin and Anfu Counties (Ji’an, China) served as the source of genomic DNA for this study. From these, thirty tick DNA samples were selected to validate the established singleplex and multiplex ddPCR assays for *Bbsl*, *B. miyamotoi*, SFGR, and *C. burnetii*, including the positive samples of early PCR screening.

More than 130 serum samples were collected from patients at Ji’an Central Hospital (Ji’an, China), following the patient selection criteria of the Lyme disease diagnostic standard (T/CPMA 007-2020) [17]. Thirty samples were selected to validate the established singleplex and multiplex ddPCR assays for *Bbsl*, *B. miyamotoi*, SFGR, and *C. burnetii*, including the positive samples of early PCR screening.

All DNA extracts were quantified and assessed for purity (NanoDrop; A260/A280: 1.8–2.0, concentration > 20 ng/μL) and integrity (agarose gel electrophoresis; no significant degradation), meeting the required specifications for ddPCR.

### 2.5. Singleplex and Multiplex ddPCR Assay Development and Optimization

To establish the multiplex ddPCR assay, singleplex reactions for each target pathogen (*Bbsl, C. burnetii*, SFGR, and *B. miyamotoi*) were first developed. These 20 µL reactions contained 10 µL of ddPCR Supermix for Probes (no dUTP), 1.8 µL of each primer (10 µM), 0.6 µL of the corresponding probe (10 µM), and 1 µL of template DNA, using a thermal profile of 95 °C for 10 min, 45 cycles of 94 °C for 30 s and 55 °C for 50 s, and a final 98 °C for 10 min. Subsequently, a multiplex system was constructed by combining all primer and probe sets into a single reaction. To enable the simultaneous detection of four targets on the two-channel QX200 system, FAM- and VIC-labeled probes were employed. The concentrations of each primer/probe set and the annealing temperature (tested across a 50 °C to 63 °C gradient) were systematically optimized. The effectiveness of the final multiplex assay was evaluated based on the two-dimensional droplet plot visualization and the quantification of positive droplets.

### 2.6. Specificity Testing of the Multiplex ddPCR Assay

The specificity of multiplex ddPCR was validated using the established multiplex ddPCR approach to detect DNA from a mixture of four pathogenic organisms (*Bbsl*, *B. miyamotoi*, *C. burnetii*, SFGR), as well as *Leptospira spirochetes*, *Treponema pallidum*, *Brucella melitensis*, *Bartonella henselae*, *A. phagocytophilum*, and *E. chaffeensis*. ddH2O was used as the negative control.

### 2.7. Valuation of Multiplex ddPCR Performance

To evaluate the assay’s performance, recombinant plasmids containing the target sequences were used. Starting from a stock concentration of 4 ng/μL, a ten-fold serial dilution series was prepared. For the linearity assessment, mixtures containing all four plasmids were prepared at concentrations corresponding to approximately 10^4^, 10^5^, 10^6^, and 10^9^ copies per 20 μL reaction. This range was selected to encompass the expected low-end sensitivity to the upper limit of the dynamic range. Each concentration was tested in triplicate. The linearity was evaluated by plotting the measured mean copy number against the expected copy number and calculating the coefficient of determination (R^2^). For the precision (reproducibility) assessment, plasmid mixtures at three concentrations spanning the middle of the dynamic range (approximately 10^5^, 10^6^, and 10^7^ copies per 20 μL reaction) were analyzed. Intra-assay precision was determined by testing each concentration in three replicate wells within a single run. Inter-assay precision was evaluated by repeating the entire experiment independently on three different days. For both assessments, the mean copy number and the coefficient of variation (CV) were calculated.

### 2.8. Simulated and Actual Sample Testing

#### 2.8.1. Simulated Sample Testing

Three tubes of simulated samples were prepared by adding 1 μL (10^5^ to 10^7^) of each of the four pathogenic bacteria to 6 μL of healthy human serum to simulate the serum samples of mixed-infected patients, and an additional tube of healthy human serum was prepared as a negative control. Detection was performed using multiple ddPCR to assess the detection rate of the simulated samples.

#### 2.8.2. Actual Sample Testing

A Qiagen DNA extraction kit was used to extract DNA from 30 tick samples and 30 serum samples. The extracted DNA was detected using qPCR, singleplex ddPCR, and multiple ddPCR, respectively. The results were compared and evaluated.

#### 2.8.3. Statistical Analysis

The agreement between singleplex and multiplex ddPCR results was evaluated using Cohen’s kappa (κ) statistic. To compare the detection rates between ddPCR (singleplex and multiplex) and qPCR for the three common pathogens (*Bbsl*, *C. burnetii*, SFGR, and *B. miyamotoi*), McNemar’s test was applied to paired sample-pathogen events, with the odds ratio (OR) and its 95% confidence interval (CI) calculated to quantify the strength of association. A *p*-value of less than 0.05 was considered statistically significant for all tests.

## 3. Results

### 3.1. Establishment of the Optimized Multiplex ddPCR Assay

Following optimization, the final reaction conditions for the multiplex ddPCR assay were determined. The optimal 20 µL reaction mixture comprised: 10 µL ddPCR Supermix, 0.36 µL each of the *Bbsl* and SFGR primers (50 µM) with 0.12 µL of their corresponding probes, 0.36 µL each of the *C. burnetii* and *B. miyamotoi* primers (50 µM) with 0.15 µL of their corresponding probes, and 2 µL of DNA template. The optimal thermal cycling conditions were established as 95 °C for 10 min, 45 cycles of 94 °C for 30 s and 55 °C for 60 s, and a final 98 °C for 10 min.

**Figure 1:**
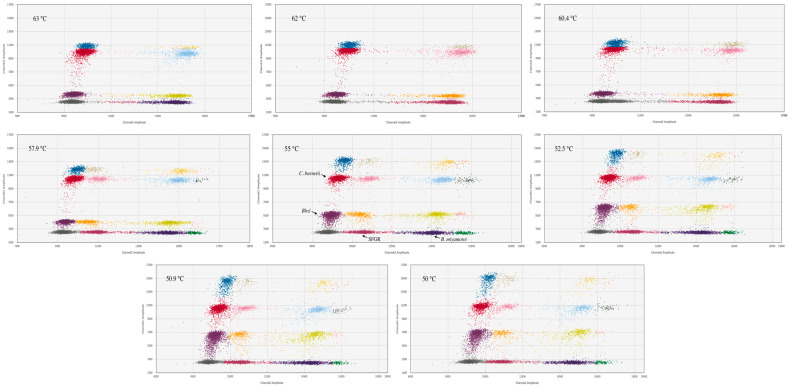
Optimization results of the annealing temperature of multiplex ddPCR.

Under these optimized conditions, the assay demonstrated efficient amplification. Analysis of the droplet data revealed that an annealing temperature of 55 °C yielded the highest target copy numbers and fluorescence signal intensity. Critically, the positive droplet clusters for all four targets were well-defined, concentrated, and clearly partitioned from one another, indicating specific amplification with minimal cross-talk (Figure 1).

### 3.2. Multiplex ddPCR Specificity Test Results

**Figure 2:**
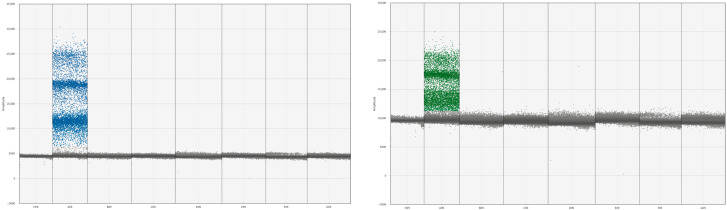
Multiplex ddPCR specificity test results.

The established multiplex ddPCR approach was used to detect the DNA of a mixture of four pathogenic bacteria (*Bbsl*, *C. burnetii*, SFGR, and *B. miyamotoi*), along with *Leptospira spirochetes*, *Treponema pallidum*, *Brucella melitensis*, *Bartonella henselae*, *A. phagocytophilum*, and *E. chaffeensis*. The established ddPCR assay demonstrated specific amplification only for the detection of *Bbsl*, *C. burnetii*, SFGR, and *B. miyamotoi*, with no non-specific amplification observed for the detection of other pathogens (see Figure 2).

### 3.3. Validation of Multiplex ddPCR Performance

**Figure 3:**
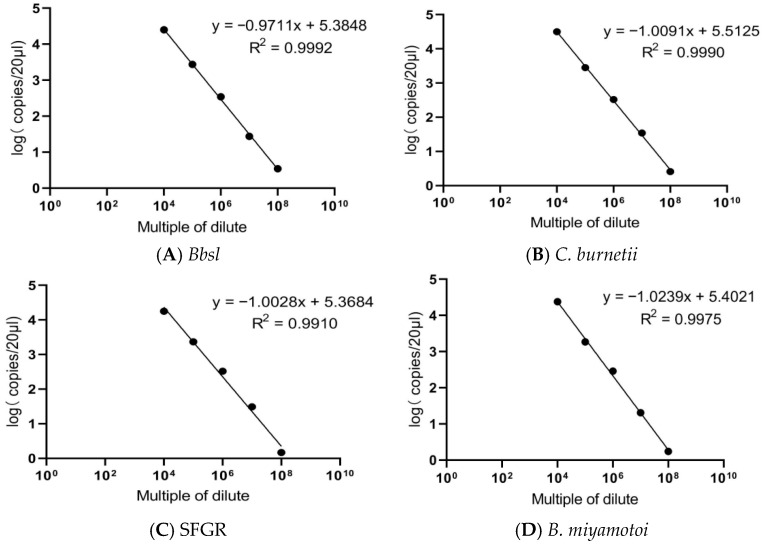
Dynamic detection range of multiplex ddPCR.

The R^2^ values of mixed samples with concentrations of 10^4^ to 10^9^ were as follows: *Bbsl* (R^2^ = 0.9992), *C. burnetii* (R^2^ = 0.9990), SFGR (R^2^ = 0.9910), and *B. miyamotoi* (R^2^ = 0.9975). These results indicate that multiplex ddPCR has an excellent dynamic range of detection (see Figure 3). The detection limits for multiplex ddPCR were as follows: *Bbsl* (4 copies/μL), *C. burnetii* (3 copies/μL), SFGR (2 copies/μL), and *B. miyamotoi* (2 copies/μL). Intra-group and inter-group repeatability tests were conducted for three concentration gradients ranging from 10^5^ to 10^7^ using the established multiplex ddPCR approach. The coefficient of variation for intra-group and inter-group repeatability experiments was less than 25% (See Table 2, Table 3, Table 4 and Table 5).

### 3.4. Detection Results from Simulated and Actual Samples

#### 3.4.1. Results of Simulated Sample Testing

All three tubes of simulated serum samples containing mixed infections of *Bbsl*, *C. burnetii*, SFGR, and *B. miyamotoi* tested positive by qPCR, singleplex ddPCR, and multiplex ddPCR.

#### 3.4.2. Results of Actual Sample Testing

qPCR, singleplex ddPCR, and multiplex ddPCR were used to analyze 30 ticks and 30 serum samples. In tick samples, qPCR detected 5, 2, and 4 positives for *Bbsl*, *C. burnetii*, and SFGR, respectively. Both ddPCR formats each detected 9 (*Bbsl*), 5 (*C. burnetii*), 7 (SFGR), and 2 (*B. miyamotoi*) positives, showing 100% agreement between them (Table 6).

In serum samples, qPCR detected 3 (*Bbsl*), 1 (*C. burnetii*), 3 (SFGR), and 4 (*B. miyamotoi*) positives, including two mixed infections. Both ddPCR formats each detected 5 (*Bbsl*), 1 (*C. burnetii*), 4 (SFGR), and 6 (*B. miyamotoi*) positives, also with 100% observed agreement (Table 6).

#### 3.4.3. Statistical Comparison

For the three pathogens common to both platforms, ddPCR demonstrated a significantly higher detection rate than qPCR in tick samples (23.3% vs. 12.2%; McNemar’s χ^2^ = 8.10, *p* = 0.004; OR = 21.0, 95% CI: 2.64–∞). In serum samples, the detection rate was also higher for ddPCR (11.1% vs. 7.8%), although this difference was not statistically significant (χ^2^ = 0.75, *p* = 0.386; OR = 3.00, 95% CI: 0.32–28.24). The multiplex ddPCR assay provided substantial added diagnostic value by detecting *B. miyamotoi* and resolving more complex co-infection profiles in both sample types.

## 4. Discussion

This study successfully developed and validated a novel multiplex droplet digital PCR (ddPCR) assay for the simultaneous detection and absolute quantification of four major tick-borne pathogens: *Bbsl*, *C. burnetii*, SFGR, and *B. miyamotoi*. Targeting the *recA*, *com1*, *ompA*, and *glpQ* genes, respectively, the assay demonstrated high sensitivity (with limits of detection ranging from 2 to 4 copies/20 μL), a broad dynamic range (over five orders of magnitude), and excellent specificity. Its performance was confirmed using both spiked and clinical samples, where it showed perfect concordance with singleplex ddPCR and a superior detection rate compared to conventional quantitative PCR (qPCR). These findings validate this multiplex assay as a robust, sensitive, and reproducible tool capable of identifying low-abundance pathogens and mixed infections in a singleplex reaction, offering significant advantages in diagnostic efficiency, cost-effectiveness, and sample consumption.

Our results align with the growing utility of multiplex ddPCR in vector-borne disease diagnostics. For instance, a previously reported broad-spectrum assay targeting *Babesia*, *Bartonella*, and *Borrelia* species underscores the value of this technology for concurrent multi-pathogen screening [18]. However, a key distinction lies in the strategic focus of each assay. The prior work aimed for extensive genus-level coverage suitable for surveillance, while our study demonstrates that multiplex ddPCR can be optimally configured for extreme sensitivity and precise quantification of a focused, clinically critical panel of bacterial pathogens. This design directly addresses the documented diagnostic challenge of detecting co-infections involving *Bbsl* with other agents such as *B. miyamotoi* and *C. burnetii*, particularly at low bacterial loads. Together, these approaches highlight the versatility of the ddPCR platform, which can be tailored either for comprehensive pathogen screening or for the highly sensitive, quantitative diagnosis of specific high-priority pathogen combinations, thereby enriching the molecular toolkit for managing complex tick-borne illnesses.

This study has several limitations. First, the clinical validation was performed with a relatively modest sample size. Future large-scale, multi-center studies are warranted to further establish the assay’s clinical utility across diverse geographical regions and patient populations. Second, although the current panel focuses on four key pathogens, integrating additional prevalent tick-borne agents without compromising sensitivity would enhance its utility for syndromic testing approaches. Furthermore, the potential impact of variables such as sample type and DNA extraction efficiency on absolute quantification merits further investigation.

## 5. Conclusions

In conclusion, we have developed and validated a novel multiplex ddPCR assay for the simultaneous and absolute quantification of *Bbsl*, *C. burnetii*, SFGR, and *B. miyamotoi*. The assay exhibits high sensitivity, a broad dynamic range, and excellent specificity. It demonstrated superior detection capability compared to qPCR and perfect agreement with singleplex ddPCR, proving particularly valuable for identifying low-abundance targets and mixed infections in a single reaction. This robust tool offers significant improvements for the clinical diagnosis and epidemiological surveillance of complex tick-borne diseases. Future work involving larger sample sets and an expanded pathogen panel is recommended to further enhance its application.

## Figures and Tables

**Table 1:** Multiple droplet digital PCR primer and probe sequences.

Bacteria	Target Gene	Primer Name	Sequence (5′-3′)	Size (bp)	Reference
*Borrelia burgdorferi sensu lato*	*recA*	*recA*-F	AGGTGGGATAGCTGCTTTTATTGAT	83	[13]
		*recA*-R	GTTCTGCAACATTAACACCTAAAGCTT		
		*recA*-P	ACAGGATCAAGAGCATG		
*Borrelia miyamotoi*	*glpQ*	*glpQ*-F	GACCCAGAAATTGACACAACCA	102	[14]
		*glpQ*-R	GGCGTAATATCGTCCGTTTTCTC		
		*glpQ*-P	AAATGTTGCACAATTATTTCCCAATCGAGC		
spotted fever group Rickettsia	*ompA*	*ompA*-F	GTTGGCAATAATAATTGGAATG	79	[15]
		*ompA*-R	CACCACCGTAAGTAAATG		
		*ompA*-P	CCGCCAGCAGGAGTACCATTA		
*Coxiella burnetii*	*com1*	Cb-F	AAAACCTCCGCGTTGTCTTCA	132	[16]
		Cb-R	GCTAATGATACTTTGGCAGCGTATTG		
		Cb-P	AGAACTGCCCATTTTTGGCGGCCA		

**Table 2:** Results of multiplex ddPCR for intra-group and inter-group repeatability tests for *Bbsl*.

	Inter-Group			Intra-Group		
Dilution Gradient	Mean Number of Copies (Copies/20 μL)	SD	RSD%	Mean Number of Copies (Copies/20 μL)	SD	RSD%
10^5^	13,072.00	378.17	2.89%	14,150.67	535.50	3.78%
10^6^	1536.00	91.65	5.97%	1715.33	163.17	9.51%
10^7^	177.67	21.01	11.82%	224.33	3.21	1.43%

**Table 3:** Results of multiplex ddPCR for intra-group and inter-group repeatability test for *C. burnetii*.

	Inter-Group			Intra-Group		
Dilution Gradient	Mean Number of Copies (Copies/20 μL)	SD	RSD%	Mean Number of Copies (Copies/20 μL)	SD	RSD%
10^5^	5749.33	200.34	3.48%	5200.67	436.10	8.39%
10^6^	539.67	56.37	10.44%	427.00	31.58	7.39%
10^7^	56.20	4.33	7.70%	72.47	14.97	20.66%

**Table 4:** Results of multiplex ddPCR for intra-group and inter-group repeatability test for SFGR.

	Inter-Group			Intra-Group		
Dilution Gradient	Mean Number of Copies (Copies/20 μL)	SD	RSD%	Mean Number of Copies (Copies/20 μL)	SD	RSD%
10^5^	3151.67	683.92	21.70%	6065.33	523.49	8.63%
10^6^	1101.33	31.07	2.82%	737.00	26.51	3.60%
10^7^	112.77	18.86	16.73%	121.67	5.13	4.22%

**Table 5:** Results of multiplex ddPCR for intra-group and inter-group repeatability tests for *B. miyamotoi*.

	Inter-Group			Intra-Group		
Dilution Gradient	Mean Number of Copies (Copies/20 μL)	SD	RSD%	Mean Number of Copies (Copies/20 μL)	SD	RSD%
10^5^	9098.33	391.74	4.31%	9845.00	688.18	6.99%
10^6^	1319.00	89.84	6.81%	880.33	78.77	8.95%
10^7^	140.33	6.11	4.35%	137.00	13.53	9.87%

**Table 6:** Results of 30 tick samples and 30 serum assays.

Sample Type	Bacteria	qPCR		Singleplex ddPCR		Multiplex ddPCR	
		Positive (Per Copy)	Detection Rate	Positive (Per Copy)	Detection Rate	Positive (Per Copy)	Detection Rate
30 ticks	*Bbsl*	5	17%	9	30%	9	30%
	*C. burnetii*	2	7%	5	17%	5	17%
	SFGR	4	13%	7	23%	7	23%
	*B. miyamotoi*	0	0%	2	7%	2	7%
30 serums	*Bbsl*	3	10%	5	17%	5	17%
	*C. burnetii*	1	3%	1	3%	1	3%
	SFGR	3	10%	4	13%	4	13%
	*B. miyamotoi*	4	13%	6	20%	6	20%

## Data Availability

The data presented in the study are available from the corresponding author upon reasonable request.
